# Molecular mechanisms of long ncRNAs in neurological disorders

**DOI:** 10.3389/fgene.2014.00048

**Published:** 2014-03-04

**Authors:** Dubravka Vučićević, Heinrich Schrewe, Ulf A. Ørom

**Affiliations:** ^1^Otto Warburg Laboratory, Max Planck Institute for Molecular GeneticsBerlin, Germany; ^2^Department of Developmental Genetics, Max Planck Institute for Molecular GeneticsBerlin, Germany

**Keywords:** neurological disorders, long non-coding RNA, protein-RNA interaction, ncRNA, brain development

## Abstract

Long non-coding RNAs (ncRNAs) have added an unexpected layer of complexity in the regulation of gene expression. Mounting evidence now links long ncRNAs to fundamental biological processes such as development and differentiation, and recent research shows important involvement of long ncRNAs in a variety of diseases including neurodegenerative disorders, such as Parkinson’s, Alzheimer’s, spinocerebellar ataxia, and Huntington’s diseases. Furthermore, long ncRNAs are speculated to be implicated in development of psychiatric disorders such as schizophrenia and bipolar disorders. Long ncRNAs contribute to these disorders in diverse ways, from regulation of transcription to modulation of RNA processing and translation. In this review, we describe the diverse mechanisms reported for long ncRNAs, and discuss how they could mechanistically be involved in the development of neurological disorders.

## INTRODUCTION

Recent technological advances such as next generation sequencing have revealed pervasive transcription of mammalian genomes (Djebali et al., 2012). It has been reported that, whereas only a small fraction of the human genome codes for proteins, 60% is being transcribed into transcripts without protein coding capacity (Derrien et al., 2012; Djebali et al., 2012). The majority of these transcripts are referred to as long non-coding RNAs (ncRNAs). The transcripts are often annotated as such judged by the lack of an appreciable open reading frame (Derrien et al., 2012).

Although only a very small fraction of annotated long ncRNAs has been well characterized, these examples show an involvement at every level of the gene expression program (Ulitsky and Bartel, 2013). Long ncRNAs have been reported to occur both as spliced, polyadenylated, and capped transcripts often transcribed by RNA polymerase II, resembling mRNAs in their physical structure (Derrien et al., 2012), and to be non-polyadenylated single-exon transcripts often involved in enhancer function (Orom and Shiekhattar, 2013). In the current review, we focus on the former group of long ncRNAs, and provide an overview of their involvement in neurological disorders.

A dominating view is that long ncRNAs often work in complex with proteins to bring about regulatory functions (Rinn et al., 2007; Tripathi et al., 2010; Bertani et al., 2011; Gong and Maquat, 2011; Wang et al., 2011; Lai et al., 2013) emphasizing one of the areas of intensive research. Many long ncRNAs have been shown to bind and guide chromatin remodeling factors to specific *loci* in the genome (Rinn et al., 2007; Bertani et al., 2011; Wang et al., 2011). Guttman et al. (2011) speculated that long ncRNAs can provide targeted specificity of individual chromatin remodelers in different cellular settings. Long ncRNAs have also been shown to bind more chromatin remodelers at the same time to coordinate their activities (Tsai et al., 2010). In addition, there are several examples of long ncRNAs regulating expression of genes post-transcriptionally (Geisler and Coller, 2013; Ulitsky and Bartel, 2013).

A large fraction of tissue specific long ncRNAs are expressed in the brain (Derrien et al., 2012). Furthermore, the majority of brain specific long ncRNAs is specifically expressed in particular regions, cell types or even subcellular compartments (Mercer et al., 2008, 2010; Derrien et al., 2012), suggesting specific regulatory roles in subsets of specialized cells. For many of these long ncRNAs it has been shown that they are functionally implicated in brain development. Long ncRNA metastasis associated lung adenocarcinoma transcript 1 works by regulating the activity of splicing factors, and controling the expression of genes involved in synapse formation, density, and maturation (Bernard et al., 2010). Additionally, a growing number of long ncRNAs has been shown to regulate expression of genes/proteins with crucial roles in neurological disorders (see **Table 1** for an overview of long ncRNAs involved in neurological disorders reviewed here in detail).

**Table 1:** Long ncRNAs involved in neurological disorders.

Long ncRNA	Regulating	Target	Associated neurological disorder
ANRIL	Transcription	INK4b/ARF/INK4a locus	Neural system tumors
BDNF-AS	Transcription	BDNF	Huntington’s disease
ncRNA-a	Transcription	CMPK1, TAL1, AURKA	Opitz–Kaveggia syndrome
*Evf-2 *	Transcription	*Dlx5/6*	Potentially Rett-syndrome, autism, schizophrenia and epilepsy
HTTAS_v1	Transcription	HTT	Huntington’s disease
SCAANT1	Transcription	Ataxin 7	Spinocerebellar ataxia 7
116HG	Transcription	Up-regulation of many genes	Prader–Willi syndrome
ATXN8OS	mRNA processing	MBLN1	Spinocerebellar ataxia 8
17A	mRNA processing	GPR51	Alzheimer’s disease
Gomafu	mRNA processing	DISC1, ERBB4	Schizophrenia, behavioral abnormalities
BACE1-AS	mRNA stability	BACE1	Alzheimer’s disease
BC200	Translation	FMR1, PABP, HNRNPA2, SYNCRIP	Alzheimer’s disease
Antisense Uchl1	Translation	UCHL1	Parkinson’s disease

## LONG ncRNAs REGULATE TRANSCRIPTION OF GENES ASSOCIATED WITH NEUROLOGICAL DISORDER

Long ncRNA antisense non-coding RNA in the INK4 locus (ANRIL) has been associated to hereditary cutaneous malignant melanoma, prostate cancer and tumors of the neural system (Pasmant et al., 2011). Furthermore, genome wide association studies have identified the ANRIL gene as a risk locus for coronary disease, intracranial aneurism, type 2 diabetes and several cancers including glioma (Pasmant et al., 2011). ANRIL is an antisense RNA transcript overlapping the INK4b/ARF/INK4a locus and participates directly in its epigenetic repression. The INK4b/ARF/INK4a locus encodes for p15, p16, and the p14ARF protein, three major players in cell fate determination (Pasmant et al., 2011). p15 and p16 are major players in the retinoblastoma (Rb) signaling pathway. Their inactivation in cells leads to inactivation of Rb, a well-studied tumor suppressor protein, and progression through the cell cycle. p14ARF activates Rb as well as the tumor suppressor p53 by promoting the degradation of MDM2. Its inactivation can also lead to cell cycle arrest (Pasmant et al., 2011). The components of the INK4b/ARF/INK4a locus are repressed by both polycomb repressive complex 1 (PRC1) and PRC2 repressive complex (Popov and Gil, 2010). Yap et al. (2010) showed that by binding to the CBX7 subunit of the PRC1 complex, ANRIL compromises its capacity to repress the INK4b/ARF/INK4a locus and control senescence in mouse embryonic fibroblasts (Pasmant et al., 2011). These data indicate that ANRIL regulates a gene locus that codes for major players involved in control of cell cycle progression and disease.

The antisense long ncRNA BDNF-AS regulates the expression of the sense strand encoded brain derived neurotrophic factor (BDNF). This protein belongs to a class of secreted growth factors that are essential for neuronal growth, maturation, differentiation, and maintenance. Its expression is impaired in neurodegenerative as well as psychiatric disorders. For example, Huntington’s disease (HD) patients have reduced levels of BDNF. Recently it was shown that knock-down of BDNF-AS resulted in upregulation of BDNF (Modarresi et al., 2012). BDNF-AS mediates its effect via PRC2. PRC2 represses gene expression through methylation of Lysine 27 of histone H3 (H3K27me2/3) by its catalytic subunit enhancer of zeste homolog 2 (EZH2) (Czermin et al., 2002). It was shown that upon knock-down of BDNF-AS the occupancy of EZH2 as well as H3K27me3 was reduced at the BDNF promoter (Vashishtha et al., 2013). Thus, BDNF-AS inhibits BDNF transcription by recruiting EZH2 to the BDNF promoter region and in that way plays an important role in the development of HD.

**FIGURE 1:**
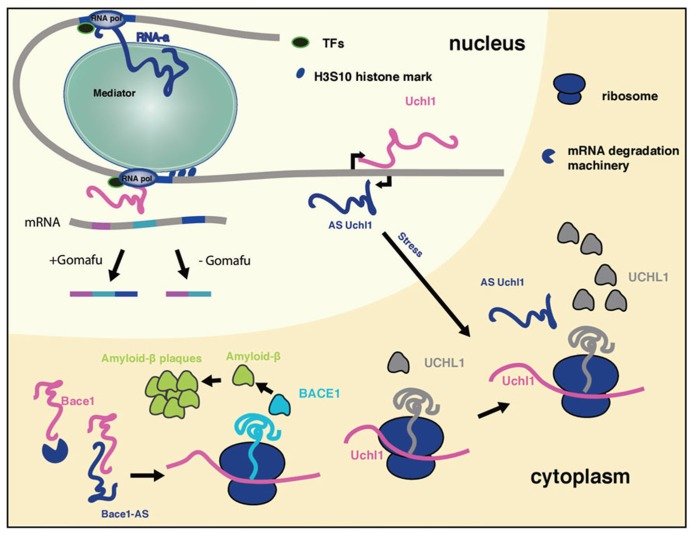
**The mechanism of long ncRNAs involved in neurological disorders**. Long ncRNAs can regulate every level of gene expression. Shown is a summary of selected long ncRNA functions discussed in the review, that take place in different cellular compartments. Due to space limitations not all long ncRNAs discussed in the review are included.

A recent study indicated that a subset of long ncRNAs, called activating long ncRNAs (RNA-a), is associated with Opitz–Kaveggia (also known as FG) syndrome, a X-linked intellectual disability syndrome, characterized by various neuronal pathologies as well as developmental abnormalities. It was shown that the Mediator complex is recruited to ncRNA-a target genes via its MED12 subunit, and regulates their expression (**Figure 1**). Mediator complexes containing missense mutant MED12 proteins corresponding to FG syndrome fail to associate with ncRNA-a (Lai et al., 2013), which might explain how these Mediator mutations can cause disease. Mediator is a evolutionary conserved multiprotein complex that controls transcription by RNA Polymerase II and acts as a key regulatory interface for the integration of activating and repressing signals at promoters and distal enhancers (Carlsten et al., 2013). The interaction of Mediator and long ncRNAs is shown to be essential for recruitment of the complex to the promoter of target genes and the H3S10 kinase activity of the Mediator complex, involved in its activating properties (Meyer et al., 2008). Loss of Mediator-ncRNA interaction might be a possible contributing factor for the neurological pathologies in FG patients. Taken together, ncRNA-a could have a prominent role in gene activation and development of FG syndrome due to its interaction with the Mediator complex.

Evf-2 is a long ncRNA that is transcribed from an ultraconserved enhancer in the Dlx-5/Dlx-6 locus that is important for proper brain development. Evf-2 regulates transcription of this unit by interacting with an activating as well as with a repressing transcription factor. Evf-2 forms *in vivo* a complex with the homeodomain containing protein Dlx-2 to activate transcription of the Dlx5/6 enhancer (Feng et al., 2006). It also recruits the repressive methylation binding protein MECP2 to the same locus. Furthermore, *Evf2* prevents CpG methylation at the Dlx-5/Dlx-6 locus, suggesting that methylated CpG sites are not responsible for MECP2 recruitment (Berghoff et al., 2013). The relationship between recruitment of MECP2 and prevention of CpG methylation by Evf2 is not clear yet and needs to be further explored. Nevertheless, loss of function of Evf2 leads to a decrease in the number of GABAergic interneurons in the early postnatal mouse hippocampus and dental gyrus. Malfunctions in GABAergic interneurons have been implicated in a number of neurological disorders including autism, schizophrenia and epilepsy (Kohtz and Berghoff, 2010). Thus, it has been speculated that Evf2 plays a role in the development of the described disorders. Additionally, malfunctions in GABAergic interneurons have been observed in Rett-syndrome, a X-linked neurological disorder affecting females 1:10,000. Mecp2 knock-out mice as a model for Rett-syndrome show extensive dysregulation of long ncRNAs (Petazzi et al., 2013). As Evf-2 appears to control the development of GABAergic interneurons, it is the subject of many studies that will hopefully help to better understand the disorders with malfunctions in these neurons and pinpoint to novel therapeutics.

Long ncRNA HTTAS_v1 is regulating the expression of Hungtiontonin (HTT) and is potentially involved in the development of HD (Chung et al., 2011). HTT is a protein that has a central role in the development of HD that is believed to be partially caused by trinucleotide repeat expansions in the gene coding for HTT. HTTAS_v1 is transcribed antisense to HTT and one of its exons includes the repeat. Overexpression of HTTAS_v1 leads to a reduction in HTT transcript levels whereas depletion leads to an increase in HTT transcript levels. This effect is dependent on the repeat length. Furthermore, transcript levels of HTTAS_v1 are reduced in frontal cortex of patients who suffer from HD, indicating that HTTAS_v1 might be an important long ncRNA contributing to the development of this neurological disorder (Chung et al., 2011).

Long ncRNA SCAANT1 is implicated in a type of polyglutamine disorder, spinocerebellar ataxia type 7 (SCA7). Spinocerebellar ataxias are a group of neurological disorders affecting the cerebellum. SCA7 is caused by CAG repeat expansion in ataxin-7 gene. Long ncRNA SCAANT1 is transcribed antisense to ataxin 7. Lack of SCAANT1 leads to an increase in ataxin 7 transcription causing a development of SCA7 in mice. Furthermore, proximal CTCF binding is required for SCAANT1 transcription. Thus, SCAANT1 acts a repressor of ataxin 7 transcription in a CTCF dependent manner and is a potential player in development of SCA7 (Sopher et al., 2011).

Long ncRNA 116HG has been shown to play a role in the development of Prader–Willi syndrome (PWS) (Powell et al., 2013). This syndrome is a neurological disorder caused by the paternal deletions of some genes on chromosome 15, including the gene that codes for the long ncRNA 116HG. Mice lacking this transcript show most of the symptoms characteristic for PWS. Long ncRNA 116HG forms a cloud in the nuclei of both mouse and human neurons (Powell et al., 2013). The cloud is formed at the site of the transcription of this long ncRNA and this ncRNA co-purifies with RBBP5 a component of mixed lineage leukemia (MLL1) activating chromatin remodeling complex. Since loss of this long ncRNA led to an up-regulation of many genes Powell and colleagues suggested that 116HG long ncRNA might act as a decoy for RBBP5 and in this way disable it to activate transcription of these genes (Powell et al., 2013). Additionally, metabolic analyses suggested that this long ncRNA regulates diurnal energy expenditure of the brain. In conclusion, long ncRNA 116HG regulates the expression of many genes potentially through interacting with RBBP5 and might help to balance energy consumption.

## LONG ncRNAs REGULATE PROCESSING OF mRNAs

ATXN8OS is a long ncRNA localized in GABAergic interneurons (Moseley et al., 2006) and plays a significant role in the development of SCA8, a type of ataxia caused by repeat expansion in ATXN8OS and ATXN8. The ATXN8OS ncRNA shares a bidirectional promoter with ATXN8 that encodes a protein known to contribute to the development of SCA8. Both the ATXN8OS and ATXN8 in SCA8 undergo a gain of function due to (CTG)_n_ repeat expansions (Moseley et al., 2006; Daughters et al., 2009). Long ncRNA transcripts with trinucleotide expansion co-localize in GABAergic neurons with the muscleblind-like splicing regulator 1 (MBLN1) and cause changes in its localization and splicing regulatory activity. As a consequence, GABA-A transporter 4 RNA undergoes alternative splicing leading to loss of GABAergic inhibition, characteristic for SCA8 (Sopher et al., 2011).

A potential contributor to the development of Alzheimer’s disease (AD) is long ncRNA 17A (Massone et al., 2011). This long ncRNA is transcribed by RNA polymerase III (Pol III) and is an antisense transcript of human G-protein-coupled receptor 51 gene (GPR51; Massone et al., 2011). Depending on alternative splicing events, this gene codes for a functional GABA B2 receptor or unfunctional GABA R2. In a human neuroblastoma cell line stable expression of long ncRNA 17A induced the production of unfunctional alternative splice isoforms for GABA R2, leading to the abolishment of GABA B2 intracellular signaling and secretion of amyloid-β peptide, characteristic for AD (Kim et al., 2013). Similarly, in cerebral cortex of AD patients 17A is upregulated and the functional GABA B2 receptor could not be detected suggesting that 17A and abolishment of GABA B2 signaling might play a role in the development of AD (Massone et al., 2011).

It has been shown that long ncRNA Gomafu (MIAT, RNBR2) plays a role in retinal cell development, brain development and post-mitotic neuronal function (Tsuiji et al., 2011; Barry et al., 2013). It localizes to specific subset of neurons in adult mice, including the CA1 region of the hippocampus and large cortical neurons. It is localized in the compartment of the nucleus enriched in splicing factors (Tsuiji et al., 2011; Barry et al., 2013). This non-coding RNA has a distinctive feature: tandem repeats of UACUAAC, a conserved intron branch point that binds to the SF1 splicing factor (Tsuiji et al., 2011). Gomafu also binds directly two additional splicing factors QKI and SRSF1. Dysregulation of this long ncRNA leads to alternative splicing patterns of DISC1 and ERBB4 (**Figure 1**). These alternative splicing patterns are similar to those observed in schizophrenic disorder. Furthermore, Gomafu is dysregulated in the cortex of schizophrenic subjects. Collectively these results indicate that Gomafu may contribute to development of schizophrenia disorder (Barry et al., 2013). In addition, Gomafu is upregulated in the region of the brain involved in behavior and addiction of cocaine and heroine users, suggesting that Gomafu might also have a role in behavioral abnormalities (Albertson et al., 2006).

With the great diversity of alternative splice forms in the human genome many more examples of long ncRNAs regulating alternative splicing of both mRNAs and other RNA species should be expected to be identified and characterized soon.

## LONG ncRNAs REGULATE mRNA STABILITY

Another long ncRNA demonstrated to play a role in AD is BACE1-AS. This long ncRNAs is transcribed antisense to β-secretase-1 protein (BACE1) and regulates BACE1 mRNA stability (Faghihi et al., 2008). BACE1 is an enzyme that generates amyloid-β that clusters in amyloid plaques that are a histological hallmark of AD. Recently, a study in mouse AD model revealed that in this clustered form amyloid-β triggers the erosion of synaptic connections between neurons which are crucial for proper functioning of the brain and AD pathophysiology (Kim et al., 2013). Upon stress stimuli BACE1-AS gets upregulated and increases BACE1 mRNA stability by duplexing with BACE1 mRNA, leading to the generation of additional BACE1 enzyme and amyloid-β (**Figure 1**; Faghihi et al., 2008). The levels of BACE1-AS are elevated in subjects with AD and its *in vivo* knock-down in mouse brain led to the downregulaton of BACE1 protein levels, reduction in amyloid-β synthesis and aggregation in the brain, signifying the importance of BACE1-AS for the development of AD (Modarresi et al., 2011).

This is an example of a long ncRNA that is reported to be acting without a protein partner, and thus represents an alternative view on the mechanism of long ncRNAs. This could be a more general property of a class of long ncRNAs that should be studied more extensive in future research.

## LONG ncRNAs REGULATE TRANSLATION

BC1 in rats and BC200 in humans, are two long ncRNAs that are compartmentalized in synaptodendrites as ribonucleoprotein particles contributing to the regulation of local protein synthesis. BC200 seems to be linked to AD development; patients suffering from AD show higher expression of BC200 in the affected area of their brain (Brodmann’s area 9), compared to same aged healthy controls. Furthermore, the levels of BC200 increase with the severity of AD in this area of the brain. Additionally, in advanced stages of AD BC200 mislocalized to the perikaryon (Mus et al., 2007). BC200 has been suggested to modulate gene expression at the translational level by interacting with different proteins: fragile X mental retardation protein (a translational repressor), poly(A)-binding protein 1 (a translation initiation regulator), heterogeneous nuclear ribonucleoprotein A2 (involved in transport of mRNAs in neurons), and synaptotagmin binding cytoplasmic RNA interacting protein (also involved in mRNA transport and potentially in local protein synthesis) (Muddashetty et al., 2002; Muslimov et al., 2006; Duning et al., 2008). Overexpression, mislocalization, as well as interaction with proteins involved in local protein synthesis and trafficking in neurons suggest BC200 to be an important player in the development of AD.

A long ncRNA transcribed antisense of the mouse ubiquitin carboxy-terminal hydrolase L1 (*Uchl1*) gene can induce the translation of Uchl1. Human UCHL1 is a neuron-restricted protein that acts as a de-ubiquitinating enzyme, ubiquitin ligase or monoubiquitin stabilizer, and its inactivation was reported in both AD and Parkinson’s disease (PD) patients. Overexpression of antisense Uchl1 led to an increase in the abundance of UCHL1 protein without affecting its mRNA levels. Only a partial overlap between the long ncRNA and mRNA is required for this activity. Uchl1 mRNA localizes predominantly in the cytoplasm whereas the antisense ncRNA is enriched in the nucleus of dopaminergic neurons. When dopaminergic cells are treated with an mTOR inhibitor, antisense Uchl1 relocalizes to the cytoplasm, triggers the binding of Uchl1 mRNA to polysomes and an increase in UCHL1 protein level is observed (**Figure 1**) (Carrieri et al., 2012). Since in genetic and neurochemical models of PD, mTOR1 inhibition protects dopaminergic neurons from apoptosis it is possible that the UCHLE-ncRNA-mTOR1 interplay might be important for the development of PD.

## OTHER LONG ncRNAs THAT ARE POTENTIALLY INVOLVED IN NEUROLOGICAL DISORDERS

Many other long ncRNAs are suspected to be involved in neurological disorders. Some of them are: TUG1 is upregulated in HD patients; PINK1-AS is potentially involved in the development of PD; Sox2OT whose gene carries the important regulator of neurogenesis gene in an alternatively spliced intron might serve as a biomarker for AD since it’s expressed exclusively in early stages of AD; Ube 3a–AS has been implicated in Angelman’s syndrome (genetic disorder that causes developmental disabilities and neurological problems) since it was suggested that it might regulate the expression of Ube3a that is mutated or deleted in this syndrome; ASFMR1, FMR4, and FMR6 long ncRNAs are downregulated in neurons of patients suffering from fragile X syndrome (genetic disorder that causes a range of developmental problems including learning disabilities and cognitive impairment) but not in healthy individuals and thus might play a role in development of this disorder; DISC2 long ncRNA might contribute to the development of schizophrenia disorder since it is disrupted by a translocation in this disorder (Pastori and Wahlestedt, 2012; Fenoglio et al., 2013; Ng et al., 2013; Pastori et al., 2014). Addtionaly, Lipovich et al. (2013) identified eight human brain specific long ncRNAs whose expression is changing in an age-related manner.

Long ncRNA NEAT1_2 has been shown to contribute to the development of amyotrophic lateral sclerosis (ALS), a motor neuron disease (Nishimoto et al., 2013). One of the proteins mutated and contributing to the development of ALS are two DNA/RNA binding proteins: TAR DNA-binding protein-43 (TDP-43) and fused in sarcoma/translocated in liposarcoma (FUS/TLS; Lagier-Tourenne and Cleveland, 2009). Recently it was showed that both TDP-43 and FUS/TLS are bound by and co-localize with the long ncRNA NEAT1_2. This long ncRNA is essential for the formation of nuclear bodies called paraspeckles and was shown to be upregulated in human motor neurons in early stage of ALS (Nishimoto et al., 2013). Thus, NEAT1_2 might contribute to the development of early stage of ALS through interaction with TDP-43 and FUS/TLS.

Long ncRNAs could also be involved in the development of HD, in which long ncRNAs HAR1F and HAR1R are affected (Pollard et al., 2006). Human accelerated regions (HARs) are fast evolving non-coding sequences in the human brain often found in the proximity of neurodevelopmental genes like GATA3. It was suggested that they might potentially participate in unique human brain functions (Pollard et al., 2006). Of these the most dramatic accelerated changes were found in the HAR1 locus that codes for the two long ncRNAs HAR1F and HAR1R (Pollard et al., 2006). The expression of both can be repressed by the RE-1-silencing transcriptional factor (REST) that pathologically (in HD) translocates to the nucleus and represses important neuronal genes in neuronal cells (Johnson et al., 2010). Future studies are needed to shed light on the mechanism of HAR1 long ncRNAs and their precise contribution to the development of HD.

## PERSPECTIVES

The repertoire of diverse functions of long ncRNAs has contributed to an increased understanding of gene regulation. Long ncRNAs are involved in brain functions in both normal and diseased state, adding an additional layer of complexity to brain function. The number of long ncRNAs has been proposed to correlate with the complexity of the organism (Taft et al., 2007), and it is tempting to speculate that brain specific long ncRNAs might be evolutionary innovations that participate in human brain function.

The fact that a long ncRNA is differentially expressed in the healthy vs the disease brain or its expression correlates with a protein known to be involved in brain disorders could be due to various reasons that are unrelated to the disease or just unspecific side-effects. One way to study the functional relevance of long ncRNAs during brain development and in neurological disorders in physiological conditions is to generate mouse models with inactivated specific long ncRNA genes. Analysis of these mutant strains could demonstrate the distinct *in vivo* roles during embryonic development and disease. Further investigations of the long ncRNA mechanisms will help to better understand how the brain functions and how disorders develop, with the potential to further drug development based on manipulation of long ncRNA expression.

## Conflict of Interest Statement

The authors declare that the research was conducted in the absence of any commercial or financial relationships that could be construed as a potential conflict of interest.
